# Tattooing of the nipple-areola complex: What not to do. A case series

**DOI:** 10.1016/j.amsu.2020.05.041

**Published:** 2020-05-30

**Authors:** Marta Starnoni, Alessio Baccarani, Massimo Pinelli, Antonio Pedone, Giorgio De Santis

**Affiliations:** Department of Medical and Surgical Sciences, Division of Plastic Surgery, University of Modena and Reggio Emilia, Largo Pozzo 71, 41124, Modena, Italy

## Abstract

**Introduction:**

Reconstruction of the nipple areola complex (NAC) is the final and easier step of breast reconstruction. However, surgeons, especially if trainees, typically have not developed tattoo skills during their training. The aim of this report is to share advice developed in our clinical practice that would minimize patient complaints and complications while performing NAC tattoos.

**Methods:**

From January 2016 to May 2018, reconstruction of NAC was performed in 48 consecutive patients. Nipple reconstruction was performed initially using skin flaps and this was followed three to eight months later by NAC tattooing. We analyzed medical reports at 12 months follow-up where we usually record patient satisfaction (very satisfied, satisfied, dissatisfied) and every patient's complaint or complication.

**Results:**

Thirty-two patients (67%) were very satisfied of NAC tattooing, twelve patients (25%) satisfied, while four patients (8%) dissatisfied. Patients complained for not having involved in choosing color, areas without sufficient pigment, extreme darkness of the tattooed NAC and artificial look.

**Conclusion:**

Tattooing is a simple and safe procedure, with a high satisfaction rate. Based on our experience, despite some technical aspects have to be considered, it is a procedure that can be safely performed by plastic surgical trainees.

## Highlights

• Surgical nipple reconstruction is not an issue. NAC tattoo is an alternative.
• Patient involvement in choosing position and color of the NAC enhances acceptance.
• Manually mix colors should be chosen rather than standard ones.
• To check uniform density of pigmentation is fundamental.
• Overcorrection of the color is not necessary.

## 1 Introduction

Nipple-areolar-complex (NAC) reconstruction and tattooing are the final steps of breast reconstruction. They are fundamental to achieve a natural aesthetic and symmetric result [1,2]. However, NAC reconstruction is an underestimated component of all breast reconstructive procedure. This could be because surgical nipple reconstruction is the technically easiest step. Historically reconstruction of areola was performed through skin graft, nowadays tattoo is considered the gold standard due to advantages such as lack of donor site, absence of scarring and, a more predictable outcome [3]. Senior surgeons are not going to reinvent themselves as tattooers. For this reason, young trainees appear to be the best ones to perform this procedure.

In our institution, tattooing of the NAC is performed by plastic surgery residents and based on our experience we have developed useful recommendations for young surgeons, without great experience, to remind them what not to do while tattooing NAC.

## 2 Methods

We conducted a retrospective analysis on patients having had reconstruction of the NAC in our academic institution (Division of Plastic Surgery, Modena University Hospital, Modena, Italy), in accordance with the principles of the Declaration of Helsinki. Considering that the analysis did not involve any modification of the widely used technique of NAC tattooing, an ethical approval was not requested. Written informed consents were obtained in all patients before NAC reconstruction. Process 2018 criteria were followed [4].

From January 2016 to May 2018, reconstruction of NAC was performed in 48 consecutive patients (9 bilateral and 39 monolateral). NAC reconstruction required from six to nine months after breast reconstruction to optimize NAC positioning. Patients didn't suffer from severe co-existing medical conditions (including immunodepression, sever skin diseases, cancer) and life expectancy < 2 years which represent reasons of NAC reconstruction deferral. Patients with missing follow-up data were excluded. Nipple reconstruction was performed initially using skin flaps [5] and this was followed three to eight months later by NAC tattooing [6].

Tattoos were performed by two surgical trainees (residents of our Department of Plastic Surgery). The two residents had a 20-h experience of NAC tattooing under the supervision of a professional tattoo artist (relatively short learning curve). NAC was tattooed using micropigmentation machine (Amiea, Berlin, Germany), and pigment colors produced by the same manufacturers, according to the widely used technique [6].

We analyzed medical reports at 12 months follow-up where we usually record patient satisfaction (very satisfied, satisfied, dissatisfied) and every patient's complaint or complication.

## 3 Results

The 48 consecutive patients had an average age was 49 years with a minimum follow up of 12 months. No missing follow-up data were recorded. All patients maintained no evidence of disease status at the follow up.

Thirty-two patients (67%) very satisfied of NAC tattooing, twelve patients (25%) satisfied, while four patients (8%) dissatisfied.

We suggested to eights patients (for different reasons, from medical comorbidities to patient distress for further breast reconstruction procedures) a reconstruction of NAC only through the tattoo avoiding the nipple reconstruction; in all these cases we obtained high satisfaction from patients.

We have experienced three cases of scar nipple dehiscence after NAC tattooing. In these three cases tattoo was performed from 3 to 4 months after nipple reconstruction with skin flaps.

Two patients undergoing bilateral NAC reconstruction complained that they were not involved in choosing of the tattoo color.

The first ten patients were tattooed after topic anesthetic cream application but three of them complained of pain during the procedure which required local anesthesia. For this reason, we stopped the use of cream in favor of local infiltration.

Three patients complained that the tattooed NAC looked too different from the contralateral. In all these cases a standard color was used without any mix of colors at disposition.

In six cases, tattooed areolas showed areas without sufficient pigment requiring correction.

Four patients were brought to our attention with the complaint of extreme darkness of the tattooed NAC. We usually explained that an overcorrection is necessary considering possible fading. Nevertheless, in all cases patients remarked they would have preferred further tattooing to improve the color rather than a too dark NAC.

Two patients asked to have smoothed borders of the areolas to minimize a too artificial look. Infections were not recorded in any case

## 4 Discussion

Despite the overall satisfaction, we analyzed every patient complaint and every form of complication occurring during the follow-up in the attempt to learn from errors.

Based on our experience we have developed the following points that can be useful for young surgeons, without great experience, to remind them what not to do while tattooing NAC:

1. Surgical nipple reconstruction is not an issue. NAC tattooing allows patients to have the symmetrizing benefit of a nipple-areolar complex without surgery. In the following situations a NAC reconstruction only through the tattoo can be considered in order to reduce possibilities of difficult healing: i) severe medical comorbidities such as diabetes, severe obesity, heavy smoking, immunosuppression for chemotherapy; ii) unfavorable local conditions such as reduced subcutaneous tissue thickness, previous radiotherapy and, an history of skin flap necrosis ii) patient's will not to have nipple projection; ii) patient's anxiety and exhaustion for years-long breast reconstruction. Tattoo is fast and easy with minimal morbidity. Furthermore, a complete reconstruction of both nipple and areola is possible in only one step [7];
2. NAC tattooing might not be performed before six to nine months after surgical nipple reconstruction in patients with thin skin flaps. A complete scar stabilization is mandatory to avoid scar dehiscence and difficulty in healing (Fig. 1) [8].

**Fig. 1:**
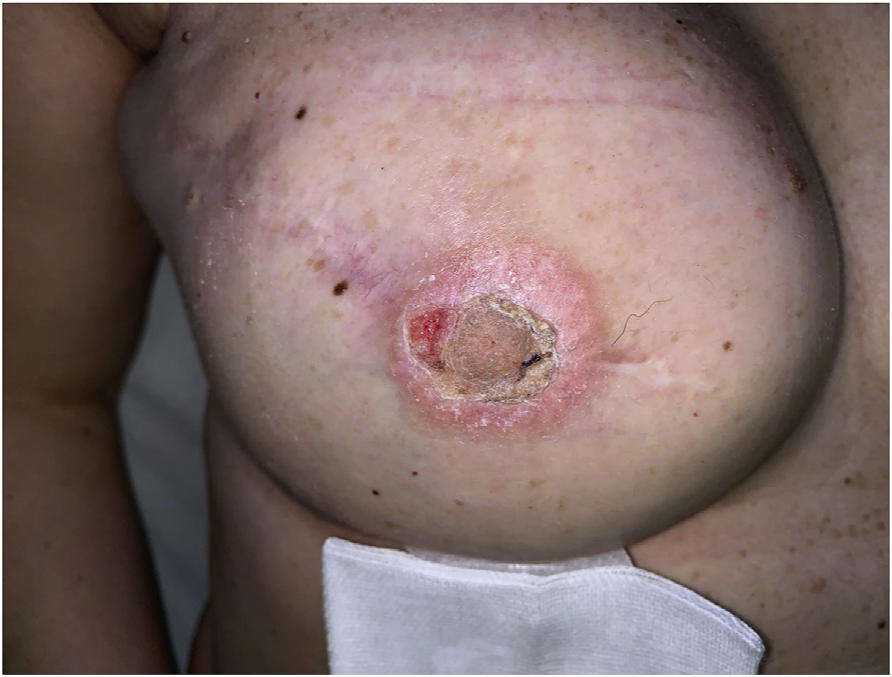
The tattoo was performed three months after the nipple reconstruction. The difficulty in healing was due to the thinness of skin. Complete healing is shown in Fig. 2.
3. Don't choose position and color of the NAC to be tattooed without patient involvement. Patient involvement enhances acceptance and compliance. Based on our experiences, in case of bilateral NAC reconstruction, we suggest to young patients a scale around the pink color while we suggest a scale around the brown color to older patients;
4. Don't use topical anesthetic cream that is time-consuming and its anesthetic effect cannot be optimal;
5. Don't use standard colors to save time but manually mix them and test the mixed color with the normal contralateral areola until they are similar (Fig. 2);

**Fig. 2:**
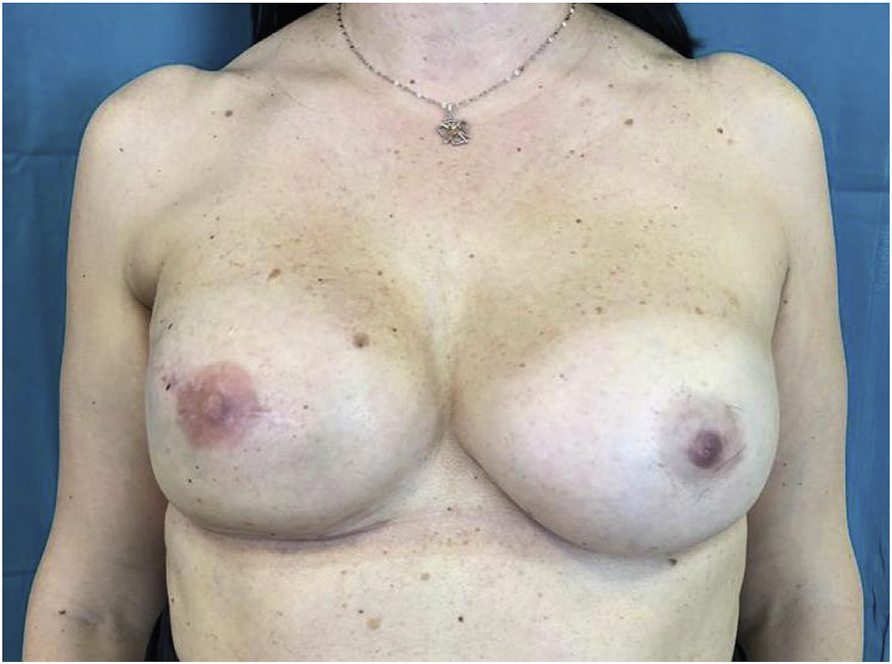
The right reconstructed NAC has a different color compared with the natural contralateral one, because color has not correctly been mixed. (For interpretation of the references to color in this figure legend, the reader is referred to the Web version of this article.)
6. Don't forget to check uniform density of pigmentation by regularly wiping the areola. A homogeneous pigmentation of the areola is essential to avoid further corrections (Fig. 3);

**Fig. 3:**
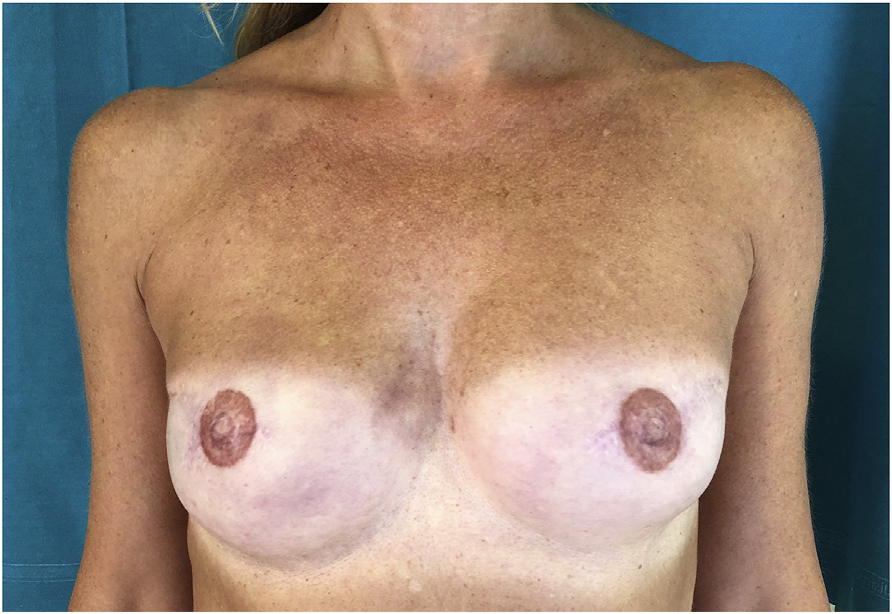
In this bilateral breast reconstruction there isn't uniform density of NACs pigmentation and a further correction is needed.
7. Overcorrection of the color by darkening the areola color for subsequent fading is not necessary. The key factor for the pigment retention is its level of deposition in the dermis (if pigment is too superficial, it will be pushed out while if it is too deep it will be removed by the macrophages). Thickness of dermis is however different from patient to patient, for this reason, it is fundamental to introduce needles into the dermis at two different depth (by placing uniform pigment at both planes of dermis);
8. Don't perform a uniform border. A gradient color will give a more natural look;
9. Prescription of systemic antibiotics or local antibiotic ointment are not necessary. We perform a sterile procedure and patient are advised to daily disinfection for 4 days postoperatively.
10. Don't use artificial light when choose the color.

In the literature numerous refinements of the technique have been proposed for consideration in view of achieving better results [7,9,10] but in our opinion these ten recommendations represent a simple way for young trainees to avoid superficial errors.

In Italy, NAC tattoo is a reimbursed procedure, and it can be performed by health-care workers in hospital or in any other health-care facility. We think that micropigmentation should be performed by well-qualified health-care workers under the supervision of a physician. In fact, breast skin after reconstruction has to be considered delicate and sensitive because of previous mastectomy and, in many cases, for the previous radiotherapy, for thin skin flaps and for the presence of a breast implant. Even if medical staff did not have professional tattooer skills, the presence of a physician is important to promptly recognize, individualize, and treat potential complications such as skin necrosis or infections [5].

## 5 Conclusions

Tattooing is a simple and safe procedure, with a high satisfaction rate. Based on our experience, despite some technical aspects have to be considered, it is a procedure that can be safely performed by plastic surgical trainees or well-qualified health-care workers under the supervision of a physician. We hope our recommendations might be useful in the future to minimize patient's complaints and to optimize NAC tattooing results.

## Ethical approval

No ethical approval needed. We describe our clinical practise.

## Sources of funding

No study sponsors.

## Author contribution

Marta Starnoni: study concept, data interpretation, writing the paper.

Alessio Baccarani: data collection.

Massimo Pinelli: data collection.

Antonio Pedone: data collection.

Giorgio De Santis: study concept, data interpretation, writing the paper.

## Registration of research studies

1. Name of the registry: Research Registry.
2. Unique Identifying number or registration ID: researchregistry5574.
3. Hyperlink to your specific registration (must be publicly accessible and will be checked): https://www.researchregistry.com/browse-the-registry#home/.

## Guarantor

Giorgio De Santis.

## Consent

Acquired written and signed consent of patients.

## Provenance and peer review

Not commissioned, externally peer reviewed.

## Declaration of competing interest

No conflict of interest.
