# Supplementary material for: Tattooing of the nipple-areola complex: What not to do. A case series
Source: Ann Med Surg (Lond). 2020 May 30;55:305–7. doi: 10.1016/j.amsu.2020.05.041 (PMC7287191; doi:10.1016/j.amsu.2020.05.041)
Supplement: Multimedia component 1 [file mmc1.pdf]

PROCESS Checklist			
**Section**	**Item**	**Checklist Description**	**Page Number**
**Title**	~~1~~	The words "case series" and the area of focus should appear in the title (e.g. disease, exposure/intervention or outcome).	
**Abstract**	~~2a~~	Introduction - what is the unifying theme of the case series.	
	~~2b~~	Methods - describe what was done, how and when was it done and by whom.	
	~~2c~~	Results - what was found.	
	~~2d~~	Conclusion - what have we learned and what does it mean	
**Introduction**	~~3~~	Explain the scientific background and rationale for the case series. What is the unifying theme - common disease, exposure, intervention and outcome, etc. Why is this study needed?	
**Methods**	~~4a~~	Registration and ethics - state the research registry number in accordance with the declaration of Helsinki - "Every research study involving human subjects must be registered in a publicly accessible database" (this can be obtained from; ResearchRegistry.com or ClinicalTrials.gov or ISRCTN). State whether ethical approval was needed and if so, what the relevant judgement reference was?	
	~~4b~~	Study design - state the study is a case series and whether prospective or retrospective in design, whether single or multi-centre and whether cases are consecutive or non-consecutive.	
	~~4c~~	Setting - describe the setting(s)and nature of the institution in which the patient was managed; academic, community or private practice setting? Location(s), and relevant dates, including periods of recruitment, exposure, follow-up, and data collection	
	~~4d~~	Participants - describe the relevant characteristics of the participants (comorbidities, tumour staging, smoking status, etc).	

		State any eligibility (inclusion/exclusion) criteria and the sources and methods of selection of participants. Describe length and methods of follow-up.	
	~~4e~~	Pre-intervention considerations e.g. Patient optimisation: measures taken prior to surgery or other intervention e.g. treating hypothermia/hypovolaemia/hypotension in burns patients, ICU care for sepsis, dealing with anticoagulation/other medications and so on.	
	~~4f~~	Types of intervention(s) deployed and reasoning behind treatment offered (pharmacological, surgical, physiotherapy, psychological, preventive) and concurrent treatments (antibiotics, analgesia, anti-emetics, nil by mouth, VTE prophylaxis, etc). Medical devices should have manufacturer and model specifically mentioned.	
	~~4g~~	Peri-intervention considerations - administration of intervention (what, where, when and how was it done, including for surgery; anaesthesia, patient position, use of tourniquet and other relevant equipment, preparation used, sutures, devices, surgical stage (1 or 2 stage, etc). Pharmacological therapies should include formulation, dosage, strength, route and duration).	
	~~4h~~	Who performed the procedures - operator experience (position on the learning curve for the technique if established, specialisation and prior relevant training).	
	~~4i~~	Quality control - what measures were taken to reduce inter or intra-operator variation. What measures were taken to ensure quality and consistency in the delivery of the intervention e.g. independent observers, lymph node counts, etc	
	~~4j~~	Post-intervention considerations e.g. post-operative instructions and place of care. Important follow-up measures - diagnostic and other test results. Future surveillance requirements - e.g. imaging surveillance of endovascular aneurysm repair (EVAR) or clinical exam/ultrasound of regional lymph nodes for skin cancer.	

**Results**	~~5a~~	Participants - reports numbers involved and their characteristics (comorbidities, tumour staging, smoking status, etc).	
	~~5b~~	Any changes in the interventions during the course of the case series (how has it evolved, been tinkered with, what learning occurred, etc) together with rationale and a diagram if appropriate. Degree of novelty for a surgical technique/device should be mentioned and a comment on learning curves should be made for new techniques/devices.	
	~~5c~~	Outcomes and follow-up - Clinician assessed and patient-reported outcomes (when appropriate) should be stated with inclusion of the time periods at which assessed. Relevant photographs/radiological images should be provided e.g. 12 month follow-up.	
	~~5d~~	Where relevant - intervention adherence/compliance and tolerability (how was this assessed). Describe loss to follow-up (express as a percentage) and any explanations for it.	
	~~5e~~	Complications and adverse or unanticipated events. Described in detail and ideally categorised in accordance with the Clavien-Dindo Classification. How they were prevented, diagnosed and managed. Blood loss, operative time, wound complications, re-exploration/revision surgery, 30-day post-op and long-term morbidity/mortality may need to be specified.	
**Discussion**	~~6a~~	Summarise key results	
	~~6b~~	Discussion of the relevant literature, implications for clinical practice guidelines, how have the indications for a new technique/device been refined and how do outcomes compare with established therapies and the prevailing gold standard should one exist and any relevant hypothesis generation.	
	~~6c~~	Strengths and limitations of the study	
	~~6d~~	The rationale for any conclusions?	

**Conclusions**	~~7a~~	State the key conclusions from the study	
	~~7b~~	State what needs to be done next, further research with what study design.	
**Additional Information**	~~8a~~	State any conflicts of interest	
	~~8b~~	State any sources of funding	
